# Skill-Specific Changes in Somatosensory Nogo Potentials in Baseball Players

**DOI:** 10.1371/journal.pone.0142581

**Published:** 2015-11-23

**Authors:** Koya Yamashiro, Daisuke Sato, Hideaki Onishi, Kazuhiro Sugawara, Sho Nakazawa, Hirofumi Shimojo, Kosuke Akatsuka, Hiroki Nakata, Atsuo Maruyama

**Affiliations:** 1 Institute for Human Movement and Medical Sciences, Niigata University of Health and Welfare, Niigata City, Japan; 2 Department of Health and Sports, Niigata University of Health and Welfare, Niigata City, Japan; 3 Department of Physical Theraphy, Niigata University of Health and Welfare, Niigata City, Japan; 4 Department of Liberal Arts, Kurume National College of Technology, Fukuoka, Japan; 5 Department of HealthSciences, Faculty of Human Life and Environment, Nara Women’s University, Nara City, Japan; The Chinese University of Hong Kong, HONG KONG

## Abstract

Athletic training is known to induce neuroplastic alterations in specific somatosensory circuits, which are reflected by changes in somatosensory evoked potentials and event-related potentials. The aim of this study was to clarify whether specific athletic training also affects somatosensory Nogo potentials related to the inhibition of movements. The Nogo potentials were recorded at nine cortical electrode positions (Fz, Cz, Pz, F3, F4, C3, C4, P3 and P4) in 12 baseball players (baseball group) and in 12 athletes in sports, such as track and field events and swimming, that do not require response inhibition, such as batting for training or performance (sports group). The Nogo potentials and Go/Nogo reaction times (Go/Nogo RTs) were measured under a somatosensory Go/Nogo paradigm in which subjects were instructed to rapidly push a button in response to stimulus presentation. The Nogo potentials were obtained by subtracting the Go trial from the Nogo trial. The peak Nogo-N2 was significantly shorter in the baseball group than that in the sports group. In addition, the amplitude of Nogo-N2 in the frontal area was significantly larger in the baseball group than that in the sports group. There was a significant positive correlation between the latency of Nogo-N2 and Go/Nogo RT. Moreover, there were significant correlations between the Go/Nogo RT and both the amplitude of Nogo-N2 and Nogo-P3 (i.e., amplitude of the Nogo-potentials increases with shorter RT). Specific athletic training regimens may induce neuroplastic alterations in sensorimotor inhibitory processes.

## Introduction

Motor skill training regimens induce neuroplastic alterations in cortical areas associated with sensory, motor and cognitive tasks. For example, performance in sports is improved by training, which develops relevant sensory–motor skills, and these improved skills are reflected in neuroplastic alterations in relevant cortical regions. Studies on athletes using somatosensory evoked potentials (SEPs) and event-related potentials (ERPs) following tactile stimulation suggest that specific training can modify the excitability of the somatosensory cortex and neuronal circuits of the brain related to specific cognitive processes[1,2,3,4].

Our previous study investigated the effect of skill-specific training on somatosensory information processing by comparing responses in stimulating the right index finger of the dominant hand in a baseball group and a sports group (swimming, track and field and soccer) that does not require fine somatosensory discrimination or motor control of the hand[1]. The results showed that peak P100 and N140 latencies and simple reaction time (SRT) were significantly shorter in the baseball group than those in the sports group. Moreover, there were significant positive correlations between SRT and both peak P100 and peak N140 latencies, suggesting that shorter SRT results from faster somatosensory information processing due to neuroplastic alterations. At the same time, baseball players are also required to stop the movement in baseball batting as quickly as possible. This processing is called ‘inhibition’ and actively suppresses the motor command to execute actions. Therefore, we can assume that skill-specific training improves inhibitory process in baseball players. However, it is very difficult to evaluate these invisible abilities; therefore, Go/Nogo paradigms have been combined with neurophysiological and neuroimaging techniques, including electroencephalography (EEG), magnetoencephalography (MEG) and functional magnetic resonance imaging (fMRI) (for review, see Nakata et al., 2014).

In a previous behavioral Go/Nogo study, Kida et al [5] investigated the effect of experience or skill level on visual SRT and Go/Nogo reaction time (Go/Nogo RT) in 82 university students (22 baseball players, 22 tennis players and 38 non-athletes) and 17 professional baseball players. The results showed that Go/Nogo RT was shorter depending on skill levels, and 2 years of batting practice in 94 senior high school students improved only Go/Nogo RT but not SRT. These findings suggest that specific training can alter task-related behavioural functions through neuroplastic changes. In a study of baseball players using EEG, Nakamoto et al. [6] examined Go/Nogo RTs and ERPs in three Go/Nogo conditions with different stimulus-response relations. Results showed that the amplitude of P300 at Fz in baseball players was larger in spatial conditions with baseball batting-specific S-R mapping than that in other no batting-specific conditions. In addition, baseball players had shorter Go/Nogo RT in baseball batting-specific S-R mapping than that in other no batting-specific conditions. They suggested that the facilitation of Go/Nogo RT in the batting-specific S-R mapping condition is likely due to a faster response selection and stronger inhibition due to task-specific training. As described above, several studies have found increased Nogo potentials and facilitation of Go/Nogo RT in baseball players using visual stimulation.

Nogo potentials have been investigated mainly using visual and auditory stimuli. Recently, somatosensory [7,8,9,10] and noxious [11] Nogo potentials have been investigated by Nakata’s group who found enhanced Nogo-related components Nogo-N140 (N2) and Nogo-P300 (P3). They suggested that Nogo potentials were not dependent on sensory modality and reflected common neural activities specific to inhibitory responses. To the best of our knowledge, although somatosensory processing is vital for both movement execution and inhibition, no studies have investigated the relationship between somatosensory Go/Nogo RT and Nogo potentials in baseball players. Moreover, previous study has shown only an enhancement of P300 in baseball players. However, the latency of P300 appears too late to reflect inhibitory processes. Therefore, we expected it to be an effect of training on earlier inhibitory component relating to response inhibition.

To test these relationships, we compared somatosensory Nogo potentials and Go/Nogo RTs of baseball players (baseball group) with those of athletes in other sports (such as track and field events and swimming) that do not require response inhibition for training and performance (sports group). We hypothesised that if specific training alters sensory processing and/or inhibitory process in neural circuits associated with the trained limb(s), Go/Nogo decisions and execution should be faster in the trained group.

## Methods

### 2.1. Subjects

24 healthy male undergraduate or graduate university students participated in this study. Written informed consent was obtained from each subject after experimental nature of the study was fully explained. Twelve subjects had played baseball for more than 9 years (baseball group), whereas the other twelve had performed other sports, such as track and field events and swimming (sports group). The baseball group and sports group were matched for age (mean, 21.2 ± 0.8 years vs. 22.7 ± 3.4 years) and height (mean, 172.8 ± 6.9 cm vs. 170.5 ± 4.6 cm). The study was conducted in accordance with the Declaration of Helsinki and approved by the ethics committee of Niigata University of Health and Welfare, Niigata, Japan. Written informed consent was obtained from all subjects.

### 2.2. Somatosensory stimulation

Somatosensory ERPs were elicited by constant current square wave pulse (pulse duration 0.2 ms) delivered to the second and fifth digits of right hand by ring electrodes. The anode was placed at the distal interphalangeal joint and the cathode at the proximal interphalangeal joint of the corresponding digits. The second digit was stimulated for the Nogo condition and the fifth digit for the Go condition. Stimulus intensity at the second digit was fixed at three times the subject’s sensory threshold and that at the fifth digit was adjusted so that the subject felt the same sensation as at the second digit. Stimuli elicited no pain or other unpleasant sensations.

### 2.3. Go/Nogo paradigm

Subjects performed a Go/Nogo paradigm consisting of 25% Nogo trials and 75% Go trials presented in random order with 2-s inter-stimulus intervals (ISIs). One session composed of 200 trials of 50 Nogo and 150 Go trials. Two sessions were performed in each subject. In Go trials, subjects were instructed to press a button using the left second digit as fast as possible when they perceived the Go stimulation delivered to the fifth digit of the right hand.

### 2.4. Recording

A SynAmps amplifier system and scan 4.3 software (Neuroscan, El Paso, TX, USA) were used for EEG acquisition. EEG was recorded using nine scalp electrodes placed at Fz, Cz, Pz, F3, F4, C3, C4, P3 and P4 according to the 10–20 system. The left earlobe was used as a reference. Electrode impedance was maintained below 5 kΩ. EEG signals were recorded with a notch filter (50 Hz) at a sampling rate of 1000 Hz. Trials with responses exceeding ±100 μV were excluded from averaging. For the Nogo condition, 100 artefact-free trials were averaged for each subject over two sessions. The temporal window of analysis was from 100 ms before to 500 ms after stimulus onset. The 100-ms period before the stimulus was used as the baseline. Band-pass filtering was set at 0.5−60 Hz.

### 2.5. Analyses

**Fig 1:**
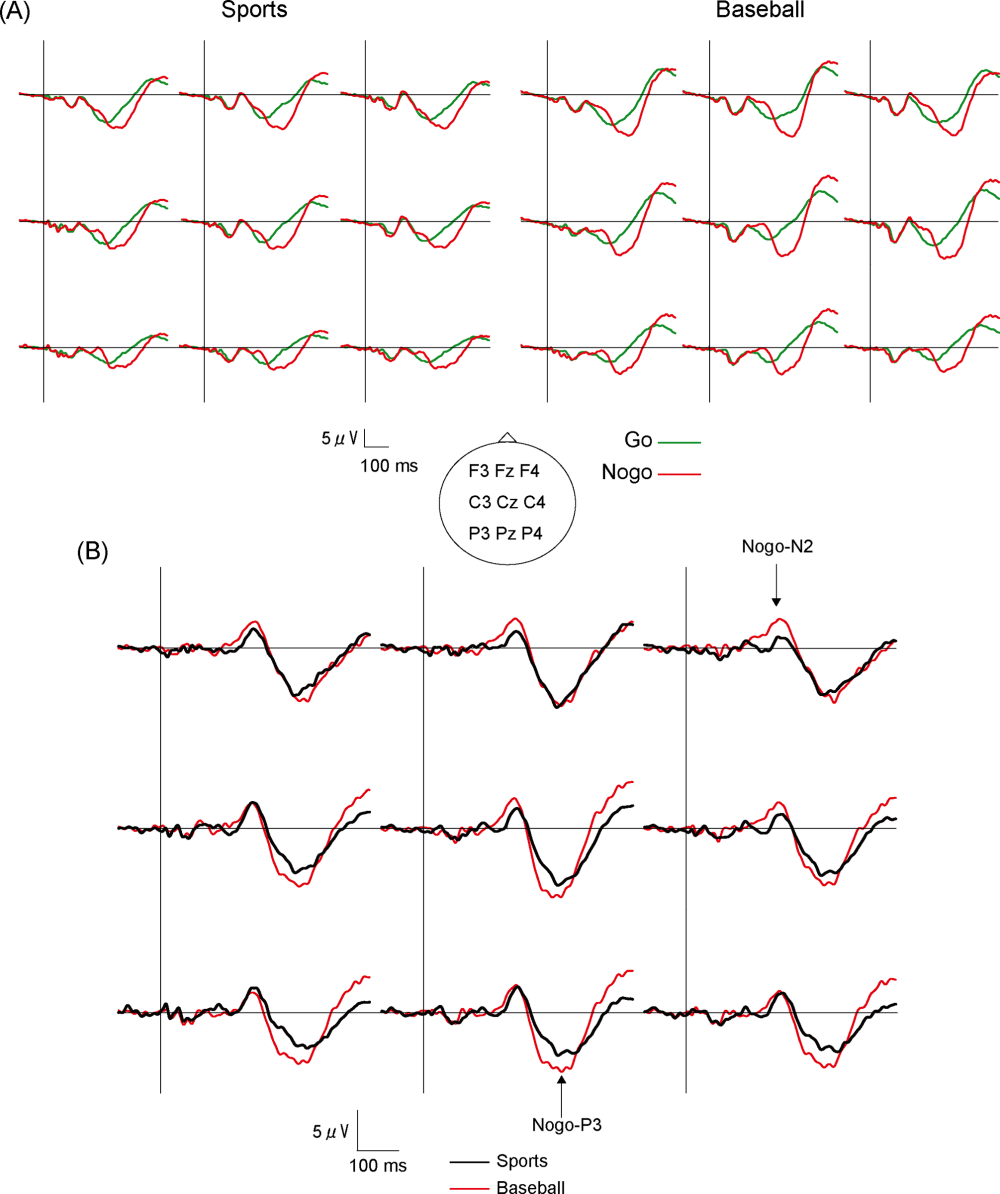
Grand averaged SEPs and Nogo potentials. Grand averaged SEPs of Go/Nogo trials (A) and subtracted (B) waveforms at nine electrodes for the sports group and the baseball group under a 75%/25% Go/Nogo paradigm.

Electrical stimulation elicited the N140 and P300 components in both Go and Nogo trials (Fig 1A). To extract Nogo potentials, we subtracted the averaged waveform of Go trials from that of Nogo trials. The subtracted waveform exhibited two prominent peaks, a negative peak at 180−250 ms after stimulus onset and a positive peak at 280−430 ms after stimulus onset named Nogo-N2 and Nogo-P3, respectively. The peak amplitudes of Nogo-N2 and Nogo-P3 were measured relative to the pre-stimulus baseline. The peak latencies and amplitudes of Nogo-N2 and Nogo-P3 were measured at all nine electrodes between 180−250 ms and 280−430 ms, respectively. Latencies and amplitudes were compared by two-way analysis of variance (ANOVA) with one within-subject factor, electrode positions (Fz, Cz, Pz, F3, F4, C3, C4, P3 and P4), and one between-subject factor, group (baseball vs. sports). The Greenhouse–Geisser epsilon was used to correct the degrees of freedom. Post-hoc tests (Bonferroni) were performed to assess pair-wise differences in peak amplitude and latency of Nogo-N2 and Nogo-P3 between the groups and electrode positions. Differences in Go/Nogo RT and commission error rate were tested by independent t-tests. Statistical significance was set at p < 0.05. Moreover, we analysed the bivariate correlations between Go/Nogo RT and both the latencies and amplitudes of Nogo-N2 and Nogo-P3 at midline electrodes (Fz, Cz, Pz).

## Results

### 3.1. Characteristics of Nogo-N2 in baseball players

Fig 1A shows the grand-averaged waveforms at all nine electrode positions for the sports group (Sports) and the baseball group (Baseball) for both Go and Nogo trials. Fig 1B presents the grand-averaged Nogo potentials for each electrode position and for both groups obtained by subtracting the specific grand-averaged Go response from the grand-averaged Nogo response. The peak latencies and amplitudes of Nogo-N2 at the nine electrode sites for both groups are shown in Table 1. Two-way ANOVA revealed a significant group × electrode interaction on Nogo-N2 amplitude (F _(2.192, 48.22)_ = 3.586, p < 0.05 ε = 0.274). Post-hoc test showed that the Nogo-N2 amplitude was significantly larger in the baseball group than that in the sports group at Fz and F4. Two-way ANOVA also revealed a significant main effect of groups on Nogo-N2 latency (F _(1, 22)_ = 7.114, p < 0.05) but no main effect of electrode position or group × electrode position interaction. The Nogo-N2 peak latency was significantly shorter in the baseball group than that in the sports group.

**Table 1:** The peak latency and amplitude ±S.D of Nogo-N2 and Nogo-P3.

	Latency (ms)				Amplitude (μV)			
	Nogo-N2		Nogo-P3		Nogo-N2		Nogo-P3	
Electrode	sports	baseball	sports	baseball	sports	baseball	sports	baseball
Fz	228 ± 11	213 ± 16	329 ± 39	320 ± 25	- 3.0 ± 1.6	- 4.4 ± 1.5*	10 ± 4.3	8.5 ± 4.2
Cz	230 ±16	216 ± 15	324 ± 36	304 ± 19	- 3.5 ± 1.5	- 4.8 ± 2.4	9.2 ± 2.7	10.1 ± 3.8
Pz	230 ± 14	220 ± 15	329 ± 37	313 ± 22	- 4.1 ± 2.1	- 4.4 ± 2.7	6.9 ± 2.3	8.6 ± 3.4
F3	232 ± 12	220 ± 11	333 ± 37	349 ± 32	- 3.2 ± 1.6	- 4.1 ± 1.4	8.8 ± 5.3	8.6 ± 4.5
C3	229 ± 12	215 ± 13	± 33	± 37	- 4.2 ± 1.4	- 4.1 ± 2.3	7.3 ± 3.3	8.6 ± 2.4
P3	231 ± 13	221 ± 15	351 ± 28	332 ± 37	- 4.0 ± 2.1	- 3.5 ± 2.1	5.6 ± 2.5	7.6 ± 2.4
F4	230 ± 14	222 ± 15	± 45	± 43	- 2.5 ± 1.7	- 4.6 ± 1.4*	9.5 ± 5.2	10 ± 4.9
C4	230 ± 14	217 ± 16	332 ± 42	327 ± 35	- 2.8 ± 2.0	- 4.6 ± 2.5	8.0 ± 3.1	8.8 ± 3.1
P4	232 ± 11	219 ± 16	337 ± 39	331 ± 36	- 3.4 ± 2.0	- 4.1 ± 2.5	6.7 ± 2.6	8.8 ± 3.0

The asterisk shows a significant group difference

*p < 0.05

### 3.2. Characteristic of Nogo-P3 in baseball players

The peak latencies and amplitudes of Nogo-P3 at nine electrode sites for both groups are also shown in Table 1. Two-way ANOVA revealed a significant difference in Nogo-P3 amplitude among electrodes (F _(1.976, 43.465)_ = 6.477, p < 0.01 ε = 0.247) but no group difference or group × electrode interaction. Post-hoc test showed that the Nogo-P3 amplitude was larger at the frontal-central area than that at the parietal area (Table 1). Two-way ANOVA revealed a significant group × electrode interaction in Nogo-P3 latency (F _(4.369, 96.121)_ = 2.674, p < 0.05 ε = 0.564). However, post-hoc tests did not detect any significant pair-wise differences.

### 3.3. Relationship between Go/Nogo RT and Nogo-N2 and Nogo-P3 components

**Fig 2:**
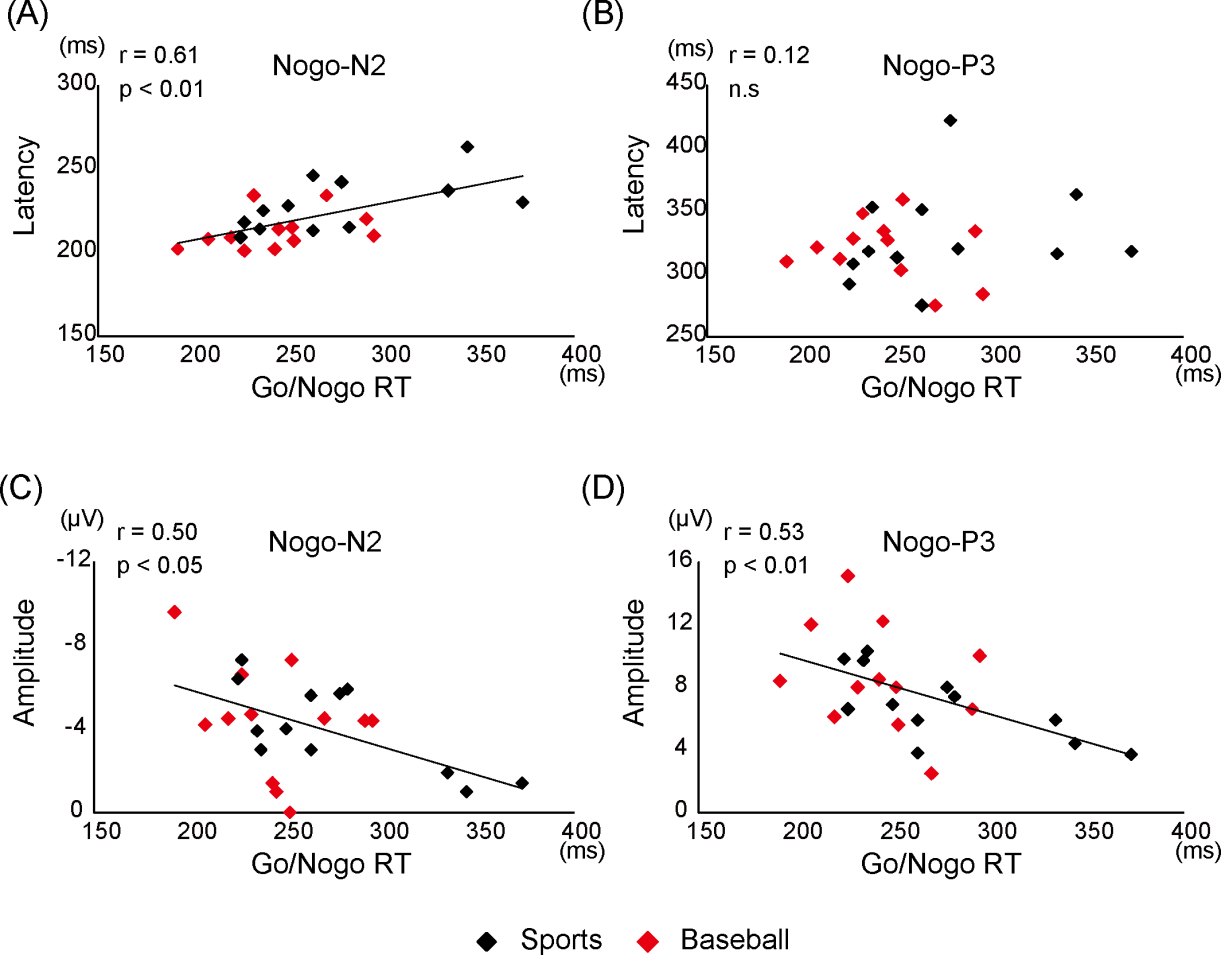
Correlations between Go/Nogo RT and Nogo potentials. Correlations between the Go/Nogo reaction time (RT) and the latencies of Nogo-N2 (A) and Nogo-P3 (B) at Fz and between the Go/Nogo RT and the amplitudes of Nogo-N2 (C) and Nogo-P3 (D) at Pz.

Go/Nogo RT was shorter in the baseball group than that in the sports group (Table 2) and independent t-tests revealed an almost significant difference (p = 0.07). Commission error rate did not show a significant difference between two groups (p = 0.63).Correlation analysis of Go/Nogo RT with Nogo-N2 and Nogo-P3 latencies across all subjects (Fig 2A and 2B, Table 3) revealed a significant positive correlation between Go/Nogo RT and N2 latency at the Fz electrode (Fig 2A, Table 3). Correlation analysis of Go/Nogo RT with across Nogo-N2 and Nogo-P3 peak amplitudes all subjects also revealed a significant correlation between Go/Nogo RT and both Nogo-N2 and Nogo-P3 at Cz and Pz (i.e. a larger amplitude Nogo-potential is associated with a short RT) (Fig 2C and 2D, Table 3).

**Table 2:** The competition history, the latency of Nogo-N2 at Fz, Go/Nogo RTs and Comission error under Go/Nogo paradigmin each subject.

	Baseball					Sports			
Subject No	Competition History(ys)	Nogo-N2 (ms)	Go/Nogo RTs (ms)	Commision Error(%)	Subject No	Competition History(ys)	Nogo-N2 (ms)	Go/Nogo RTs (ms)	Commision Error(%)
1	13	220	291	4	1	15	228	250	8
2	10	210	295	4	2	8	214	230	3
3	10	234	232	3	3	9	218	227	4
4	13	214	245	5	4	10	263	344	9
5	15	201	227	4	5	10	227	373	3
6	10	208	208	5	6	8	246	263	5
7	11	202	243	7	7	14	213	263	1
8	9	209	220	2	8	8	215	282	6
9	11	207	253	5	9	7	242	278	4
10	10	215	252	2	10	7	225	237	7
11	11	202	192	4	11	8	209	225	4
12	10	234	270	3	12	12	237	334	1
mean	11.1	213	244	4.0	mean	9.7	228	276	4.4
S.D	1.7	11.3	31.1	1.4	S.D	2.7	16.2	49.2	2.6

**Table 3:** The r values of correlations between Go/Nogo RT and the latencies of Nogo-N2 and Nogo-P3 and between Go/Nogo RT and the amplitude of Nogo-N2 and Nogo-P3 at the three midline electrodes.

	Fz	Cz	Pz
**latency**			
Nogo-N2	0.61**	0.36	0.35
Nogo-P3	0.12	0.20	0.09
**amplitude**			
Nogo-N2	0.07	0.41*	0.50*
Nogo-P3	- 0.23	-0.46*	-0.53**

*p < 0.05

**p < 0.01

## Discussion

We demonstrate that skill-specific training can alter somatosensory Nogo potentials related to response inhibition, suggesting that training induces neuroplastic alterations in both neural circuits controlling sensorimotor execution and those controlling inhibition. This conclusion was derived from a comparison of somatosensory Nogo potentials and Go/Nogo RT (related to response inhibition) of hand movements between baseball players who require frequent and rapid response inhibition of hand movements and athletes involved in other sports (track and field events and swimming) who do not require such movements. The results showed that (i) the peak latency and amplitude of Nogo-N2 were significantly shorter and larger in the baseball players than those in other athletes, (ii) there was a significant positive correlation between Go/Nogo RT and the peak latency of Nogo-N2, (iii) there were a significant correlations between Go/Nogo RT and the peak amplitude of Nogo-N2 and between Go/Nogo RT and the peak amplitude of Nogo-P3 (i.e. amplitude of the Nogo-potential increases with shorter RT). These findings suggest that skill-specific training alters inhibitory processes in the somatosensory system.

### 4.1. Shorter Nogo-N2 latency in baseball players

Neuroplastic alterations in the somatosensory cortex have been demonstrated in athletes and other professionals requiring extensive specific sensorimotor skill training [2,4,12,13,14]. Iwadate et al., [3] investigated the effects of long-term training on somatosensory ERPs by comparing soccer players with non-athletes in a lower limb-targeted oddball paradigm. The results showed that long-term training shortens the P300 latency in soccer players compared with non-athletes. Similarly, our recent study using a hand-targeted somatosensory-cued motor reaction test revealed that P100 and N140 latencies and SRT were shorter in a baseball player group than those in a sports group that did not require fine somatosensory discrimination or motor skill. Moreover, there were significant positive correlations between SRT and both P100 and N140 latencies [1].

Peak latencies are known to be important indices related to the stimulus classification speed or stimulus evaluation time in Go/Nogo paradigms [15]. In the present study, the latency of somatosensory Nogo-N2 was significantly 15 ms shorter in the baseball group than that in the sports group and consistent with previous findings that long-term specific training can alter somatosensory pathways and cognitive processes [1,3,4]. The reduction in somatosensory Nogo-N2 latency was roughly equal to the reduction in P100 and N140 latencies in our previous study. Therefore, we suggest that reduced somatosensory Nogo-N2 latency may be attributed to a general acceleration of task-specific somatosensory processing as reflected by shorter P100 and N140 latencies.

Kida et al. [5] compared the Go/Nogo RT in a visual task among baseball players of four skill-levels, low, medium, high and professional and found that the Go/Nogo RT of higher skilled baseball players was significantly shorter than that of lower skilled players, while that of professional baseball players was the shortest. They suggested that intensive practice, including Go/Nogo decision making, improved the Go/Nogo RT. They also found that the strongest correlation was between SRT and Go/Nogo RT in the professional baseball players and suggested that SRT limits the Go/Nogo RT. Moreover, Russo et al. [16] investigated neural mechanisms responsible for fencer’s fast and flexible behaviour by recording ERPs, SRTs and Go/Nogo RTs in visual modality. They founded faster Go/Nogo RT and faster brain activity as the discrimination stage started earlier in fencers than controls (150 ms vs. 200 ms) and suggested fencer’s specific ability is likely due to faster stimulus discrimination facilitated by greater attention. Similarly, there was a significant positive correlation between Go/Nogo RT and somatosensory Nogo-N2 latency in the present study. Taken together, these results indicate that skill-specific training accelerates somatosensory processing, thereby facilitating both Go and Nogo decisions.

### 4.2. Enhancement of Nogo-N2 amplitude in baseball players

We also found significant enhancement of somatosensory Nogo-N2 amplitude in the baseball player group compared with the sports group at Fz and F4, in accordance with previous athlete studies. For example, Iwadate et al. [3] found that the athlete (soccer) group demonstrated significantly larger somatosensory N140 amplitude in bilateral frontal lobes than that in the non-athlete group during upper- and lower-limb tasks and suggested that activation of the frontal cortex in athletes may be accelerated by the attentional ability associated with physical training. The term “attentional ability” here shows the active attention, such as sustained attention [17] and selective attention [18,19]. These abilities are required not only by baseball players but also by athletes who take part in open-skill games. In addition to attentional ability, baseball players are required to judge, take action or inhibit action; thus, enhancing Nogo-N2 amplitude might be a specific characteristic for baseball players. This implies that long-term specific physical training may modulate ERP components related to higher order brain functions, such as attention, decision making and response inhibition.

In a visual Go/Nogo study, Russo et al. [16] showed that fencers had larger amplitude Nogo-N2 (peak 270 ms) than controls and suggested that these athletes exhibit stronger prefrontal inhibition. However, Nakamoto et al., [6] found no difference in visual Nogo-N200 amplitude between a baseball group and a non-baseball group, although visual Nogo-N200 is analogous to somatosensory Nogo-N140 (N2). Similarly, Nakata et al. [7] found no correlation between Go/Nogo RT and somatosensory Nogo-N140 amplitude. A reasonable explanation for these differences is the analysis method used. Our study and Russo’s study extracted Nogo components by subtraction as in several previous studies [9,15,20,21].

There was also a significant correlation between Go/Nogo RT and the amplitude of Nogo-N2 in the present study (shorter Go/Nogo RT associated with higher amplitude Nogo-N2) consistent with several previous studies. For example, Jodo and Kayama [22] found that Nogo-N2 amplitude was significantly larger in the shorter RT subgroup (high response inhibition: HI) than that in the longer RT subgroup (low response inhibition: LI) in a visual Go-Nogo task and suggested that enhancement of visual Nogo-N2 amplitude reflects the activity of a cortical response inhibition system. Similarly, Band et al. [23] showed that the enhancement of visual Nogo-N2 amplitude was related to faster Go/Nogo RT. Therefore, the enhancement of somatosensory Nogo-N2 amplitude in the baseball group may reflect stronger inhibitory function, leading to shorter Go/Nogo RT.

### 4.3. Relationship between somatosensory Nogo-P3 and Go/Nogo RT

The present study revealed a significant correlation between Go/Nogo RT and the peak amplitude of Nogo-P3, i.e., shorter Go/Nogo RT was associated with larger Nogo-P3 amplitude. This finding is also consistent with previous auditory [24] and somatosensory [7] studies. Nakata et al. [7] suggested that the strength of Nogo-related neural activity increases the response speed for the Go stimulus. In addition, several studies have shown that Nogo-P3 amplitude reflects the effectiveness of inhibition [25] and strength of inhibition [6].

In contrast, P300 is thought to reflect a relatively late-stage of stimulus-related processing, such as stimulus evaluation and context update [26, 27], and the amplitude of P300 is known to be proportional to the amount of attentional resources devoted to a given task [28]. It is a matter of debate which amplitude, Nogo-N2 or Nogo-P3, better reflects response inhibition. On the basis of these findings, we speculate that Nogo-P3 reflects the amount of attention needed to stop the movement rather than the strength of inhibition. Lavric et al. [29] found differences in N2 in a visual Go/Nogo paradigm with equal Go and Nogo trial frequency using tomographic analyses and suggested that N2 reflects conflict monitoring and response inhibition. In the present study, the grand averaged somatosensory Go/Nogo RT for all subjects was 260 ms, while the averaged latencies of somatosensory Nogo-N2 and Nogo-P3 were 223 ms and 314 ms, respectively. Thus, the latency of Nogo-P3 appears too late to reflect response inhibition, supporting our speculation that Nogo-N2 is a more sensitive indicator of inhibition strength.

## Conclusion

In summary, the present study revealed alteration of the somatosensory Nogo-N2 component in baseball players. These finding suggests that long-term specific training alters not only somatosensory processing but also higher order cognitive functions, such as decision making.
