# Correction: Skill-Specific Changes in Somatosensory Nogo Potentials in Baseball Players

**DOI:** 10.1371/journal.pone.0146379

**Published:** 2015-12-30

**Authors:** Koya Yamashiro, Daisuke Sato, Hideaki Onishi, Kazuhiro Sugawara, Sho Nakazawa, Hirofumi Shimojo, Kosuke Akatsuka, Hiroki Nakata, Atsuo Maruyama

There is an error in Table 1. The values in column Nogo-P3, row C3 and F4 were listed incorrectly. Please see the corrected Table 1 here.

**Table 1:** The peak latency and amplitude ±S.D of Nogo-N2 and Nogo-P3

	Latency (ms)				Amplitude (μV)			
	Nogo-N2		Nogo-P3		Nogo-N2		Nogo-P3	
Electrode	sports	baseball	sports	baseball	sports	baseball	sports	baseball
Fz	228 ± 11	213 ± 16	329 ± 39	320 ± 25	- 3.0 ± 1.6	- 4.4 ± 1.5*	10 ± 4.3	8.5 ± 4.2
Cz	230 ±16	216 ± 15	324 ± 36	304 ± 19	- 3.5 ± 1.5	- 4.8 ± 2.4	9.2 ± 2.7	10.1 ± 3.8
Pz	230 ± 14	220 ± 15	329 ± 37	313 ± 22	- 4.1 ± 2.1	- 4.4 ± 2.7	6.9 ± 2.3	8.6 ± 3.4
F3	232 ± 12	220 ± 11	333 ± 37	349 ± 32	- 3.2 ± 1.6	- 4.1 ± 1.4	8.8 ± 5.3	8.6 ± 4.5
C3	229 ± 12	215 ± 13	327 ± 33	326 ± 37	- 4.2 ± 1.4	- 4.1 ± 2.3	7.3 ± 3.3	8.6 ± 2.4
P3	231 ± 13	221 ± 15	351 ± 28	332 ± 37	- 4.0 ± 2.1	- 3.5 ± 2.1	5.6 ± 2.5	7.6 ± 2.4
F4	230 ± 14	222 ± 15	339 ± 45	360 ± 43	- 2.5 ± 1.7	- 4.6 ± 1.4*	9.5 ± 5.2	10 ± 4.9
C4	230 ± 14	217 ± 16	332 ± 42	327 ± 35	- 2.8 ± 2.0	- 4.6 ± 2.5	8.0 ± 3.1	8.8 ± 3.1
P4	232 ± 11	219 ± 16	337 ± 39	331 ± 36	- 3.4 ± 2.0	- 4.1 ± 2.5	6.7 ± 2.6	8.8 ± 3.0

The asterisk shows a significant group difference

*p < 0.05
